# Immunologic studies in patients with malignant melanoma in Uganda.

**DOI:** 10.1038/bjc.1969.89

**Published:** 1969-12

**Authors:** J. L. Ziegler, M. G. Lewis, J. M. Luyombya, J. W. Kiryabwire


### IMMUNOLOGIC STUDIES IN PATIENTS WITH MALIGNANT MELANOMA IN UGANDA

J. L. ZIEGLER*, M. G. LEWIS, J. M. S. LUYOMBYA, AND J. W. M. KIRYABWIRE

*From the Lymphoma Treatment Centre and the Departments of Pathology and Surgery, Makerere University College Medical School, Kampala, Uganda*

Received for publication July 10, 1969

THE natural history of malignant melanoma in Uganda has been recently studied in a retrospective analysis of 200 cases, and three clinical groups have been delineated (Lewis and Kiryabwire, 1968). Group 1 (40%) consisted of patients with a relatively long history of a localised tumour, usually on the sole of the foot, with no metastases. Group 2 (48%) included patients with a primary lesion with regional or distant metastases and a short clinical history. Group 3 (12%) consisted of patients presenting with metastatic melanoma in whom no primary lesion could be found. There were no distinguishing histological features among the three groups, and the authors suggested that a difference might exist in the host response to this tumour. Lewis (1967) also demonstrated an *in vitro* cytotoxic effect of autologous serum against melanoma cells of Group 1 but not Group 2 patients, suggesting possible immunological differences.

The present study was undertaken to examine the immunologic status in the patient with localised and metastatic malignant melanoma, and to determine if defective immune mechanisms might be related to the occurrence of tumour metastasis. To this end, a study of cellular and humoral immunity in 19 Ugandan patients with malignant melanoma was performed.

### MATERIAL AND METHODS

All patients with a histopathological diagnosis of malignant melanoma admitted to the Lymphoma Treatment Centre or the surgical wards of the New Mulago Hospital, Kampala, Uganda, between January 1968 and January 1969 were studied. The patients were classified into one of the 3 clinical groups mentioned above. All patients were studied before surgery or the administration of any cytotoxic agents.

### *Cellular immunity*

Dinitrochlorobenzene (DNCB)† and 5 common skin test antigens were used to evaluate the delayed hypersensitivity response. A sensitising dose of 2000 μg. of DNCB in 0·1 ml. of acetone was applied to the medial aspect of the right upper arm within a 2 cm. polyethylene ring, allowed to evaporate, and covered with an adhesive bandage for one week (Brown *et al.*, 1967). Fourteen days following the sensitising dose, 50 μg. and 100 μg. of DNCB in 0·1 ml. acetone were similarly

* Address for reprints: John L. Ziegler, MD, Lymphoma Treatment Centre, P.O. Box 3935, Kampala, Uganda.

† (1-chloro-2,4-dinitrobenzene, Eastman Organic Chemicals, Rochester, New York).

applied to separate sites on the right forearm. Challenge tests were read at 48 hours as positive if induration, vesicles or bullae were present.

The skin tests employed were " Brucellergen " protein nucleate (Merck, Sharp and Dohme, West Point, Pa.); *Candida albicans* extract 1 : 100 (supplied as dermatophytin O, Holister-Stier Laboratories, Spokane, Wash.); mumps antigen (Eli Lilly and Company, Indianapolis, Ind.); intermediate strength purified protein derivative of tuberculin (0·0002 mg. PPD) (Parke Davis & Co., Detroit, Mich.) and trichophyton 1 : 30 (supplied as dermatophytin, Holister-Stier Laboratories, Spokane, Wash.). Skin tests were administered as 0·1 ml. intradermal injections in the left forearm and read at 48–72 hours as positive if greater than 5 mm. of induration was present.

Lymphocyte transformation with phytohaemagglutinin-M (PHA) (Difco, Detroit, Mich.) *in vitro* was determined by the following procedure. Twenty ml. of heparinised blood was allowed to settle at room temperature for 1–2 hours, and the leukocytes were counted and adjusted to a final concentration of $10^6$/ml. with Hyland agammaglobulinaemic newborn calf serum. One ml. of leukocyte suspension was incubated with 2 ml. of minimal essential media containing 100 units of streptomycin, 100 units of penicillin and 50 $\mu$g. of glutamine per ml. (Flow Laboratories, Rockville, Md.). Cultures were prepared in duplicate with and without 0·05 ml. of PHA, and incubated for 4 days at 37° C. Cultures were harvested by centrifugation at 1500 r.p.m. for 8 minutes in an International Centrifuge No. 269 head, fixed in a freshly prepared mixture of 1 : 9 glacial acetic acid and 95% alcohol for 10 minutes and recentrifuged. The cells were pipetted on slides, air dried, and stained with Giemsa's stain. For each culture 300 cell differential counts of normal lymphocytes, lymphoblastoid lymphocytes, mitoses and macrophages were performed and the percentage of transformed lymphocytes were calculated from the ratio of lymphoblastoid and mitotic cells to the total lymphoid cells counted. Average percentages of the two unstimulated and PHA-stimulated cultures were calculated separately.

*Humoral immunity*

Antibody response to Vi antigen* was measured following the intramuscular administration of 100 $\mu$g. of antigen. Pre-immunisation and 14-day post-immunisation serum was collected, stored at −20° C., and serum titres were determined in two-fold dilutions by a haemagglutination technique (Landy and Lamb, 1953).

Immunoglobulin levels were measured in duplicate by the gel diffusion method of Fahey and McKelvey (1965), using Hyland antibody-agar plates. Assays were performed at the National Cancer Institute, Bethesda, Md., on specimens of serum collected and stored at −20° C. and shipped frozen from Kampala.

*Controls*

Twelve adult males who were hospitalised for trauma served as controls for the Vi antibody response and immunoglobulin determinations (by the courtesy of Mr. John Taylor, Department of Surgery, Mulago Hospital, Kampala, Uganda). Nineteen of 20 normal Ugandan children, adolescents and young adults were

* A polysaccharide isolated from *E. coli* (5396/38), kindly supplied by Dr. M. E. Webster, National Heart Institute, and prepared by Dr. J. F. Gallelei, Clinical Center Pharmacy Dept., National Institute of Health, Bethesda, Md.

successfully sensitised with DNCB and responded with a typical indurated or vesicular reaction. Lymphocyte transformation with PHA was evaluated in 10 healthy African adults.

RESULTS

Table I shows the clinical features of the patients at the time of testing. Eight patients were Group 1, 9 patients were Group 2, and 2 patients were Group 3.

TABLE I.—*Clinical Features of Patients with Malignant Melanoma*

| Group | Patient | Age | Sex | Primary site | Metastases | Duration of symptoms (months) |
|---|---|---|---|---|---|---|
| 1 | 1 | 40 | F | R. Heel | — | 3 |
| | 2 | 50 | F | L. Foot | — | 24 |
| | 3 | 39 | F | R. Foot | — | 4 |
| | 4 | 50 | M | R. Foot | — | 12 |
| | 5 | 50 | M | L. Heel | — | 42 |
| | 6 | 33 | F | R. Foot | — | 12 |
| | 7 | 80 | M | R. Foot | — | 36 |
| | 8 | 28 | M | R. Foot | — | 24 |
| 2 | 9 | 70 | F | R. Heel | Inguinal, Lung, Liver | 18 |
| | 10 | 35 | F | L. Heel | Inguinal | 12 |
| | 11 | 50 | F | L. Foot | Inguinal | 5 |
| | 12 | 60 | F | R. Foot | Inguinal | 12 |
| | 13 | 40 | F | R. Foot | Inguinal | 24 |
| | 14 | 32 | F | R. Heel | Inguinal | 36 |
| | 15 | 45 | F | R. Foot | Inguinal, Liver, Lung, Brain | 10 |
| | 16 | 50 | M | R. Toe | Inguinal | 24 |
| | 17 | 45 | M | R. Toe | Inguinal, Lung, Skin, Bone | 3 |
| 3 | 18 | 30 | M | — | Inguinal | 6 |
| | 19 | 26 | M | — | Inguinal | 12 |

All patients but 2 (cases 18 and 19) had a primary lesion on the sole of the foot; the two exceptions had groin metastases without detectable primary lesions, although case 19 had a dark black spot on the heel which may have been a regressed primary tumour. For purposes of this study Group 3 patients are considered part of Group 2, as they manifest metastatic disease. In a number of instances intervening circumstances (death, inadvertent discharge, or running away from hospital) prevented the completion of assays requiring a 2 week interval (DNCB sensitisation and Vi antibody titre).

TABLE II.—*Delayed Hypersensitivity Response in Malignant Melanoma*

| | Skin tests | | | DNCB | | |
|---|---|---|---|---|---|---|
| | No. tested | No. positive | (%) | No. tested | No. positive | (%) |
| Group 1 | 8 | 8 | (100) | 7 | 6 | (86) |
| Groups 2 & 3 | 11 | 8 | (73) | 6 | 4 | (67) |

Table II presents the results of skin testing and DNCB sensitisation. All Group 1 patients and 73% of Group 2 patients had at least one positive intradermal test. DNCB testing revealed 86% positive responses in Group 1 and 67% positive responses in Group 2. The 3 Group 2 patients who had negative intradermal tests were not tested for DNCB reactivity (due to death, discharge or running away). The 2 negative DNCB reactors had positive skin tests. One patient in Group 1 (No. 3), who had a positive DNCB developed metastases one year following surgery, and DNCB reactivity was still present. There is no difference in skin test and DNCB reactivity between Groups 1 and 2, and the delayed hypersensitivity response in both groups appears intact.

FIG. 1.—Lymphocyte transformation in malignant melanoma. (Crossbars indicate mean ± S.D. in controls.)

FIG. 2.—Vi antibody response in malignant melanoma. (Crossbars indicate mean ± S.D. in controls.)

Per cent lymphocyte transformation with PHA *in vitro* is shown in Fig. 1, and reveals normal values in the 17 patients tested.

Antibody response to Vi antigen in 13 patients who had pre-immunisation titres of less than 1 : 8 is shown in Fig. 2. The antibody titres, expressed as increments in tube dilution, are comparable in both clinical groups, and are within the normal range.

FIG. 3.—Immunoglobulin levels in malignant melanoma. (Crossbars indicate mean ± S.D. in controls.)

Fig. 3 shows the immunoglobulin levels in Group 1 and Group 2 patients. There is no difference in immunoglobulin levels in the two groups, and with the exception of four patients with slightly elevated IgA or IgM levels, all the values are within the normal range.

### DISCUSSION

The clinical course of some cases of malignant melanoma is characterised by (1) spontaneous regressions, (2) regression of primary lesions when metastases occur, and (3) long remissions following surgical removal of tumour, which have suggested the influence of host immunological mechanisms on the clinical behaviour of the tumour. The results of this study reveal no differences in immunologic competence in patients with localised or metastatic malignant melanoma in Uganda and no evidence of immunologic impairment in either cellular or humoral responses. Thus, a generalised non-specific impairment of host immunity cannot be implicated in the dissemination of malignant melanoma from a localised site.

It is probable that tumour-specific antigenic differences may be present in the patients with Group 1 or Group 2 melanoma. This phenomenon is suggested by the study of Fass and co-workers (1969, unpublished data) who showed positive delayed hypersensitivity responses to autologous tumour extracts in Group 1 but not in Group 2 melanoma patients. Other postulated mechanisms for the loss of immunologic surveillance of tumour cells include the development of immunologic tolerance, immunoselection, or immunologic enhancement (Smith, 1968).

Some degree of depressed immunologic reactivity has been observed in patients with other types of advanced cancer (Solowey and Rapaport, 1965; Lamb *et al.*, 1962; Krant *et al.*, 1968). This phenomenon may be related to many factors, including the type and extent of cancer, the age of the patient, the nature of the antigenic stimulus, involvement of lymphatic tissue by tumour, chemotherapy, radiotherapy, intercurrent or debilitating disease, and nutritional status. The present study revealed normal immune responses in all patients with melanoma, even though some had metastatic disease. Thus all patients are presumably capable of initiating an anti-tumour immunologic response, and other factors such as tumour-specific antigenicity should be investigated to explain the containment or spread of malignant melanoma.

### SUMMARY

The natural history of malignant melanoma in Uganda suggests differences in the immune status of the host in the localised (Group 1) and disseminated (Group 2) condition. Cellular and humoral immune responses were evaluated in this study and were found to be normal in both clinical groups. Therefore, generalised non-specific suppression of immune mechanisms cannot explain these clinical differences.

The authors wish to thank Mrs. Susan Weymark, Mrs. Mary Proctor and Miss Eleanor Stashick for technical assistance and Mrs. P. Hobson and Mrs. B. Cavanaugh for preparation of the manuscript. Dr. Martin H. Cohen and Dr. Paul P. Carbone of the National Cancer Institute contributed to the design of the study and coordinated immunoglobulin determinations at the National Institutes of Health. The Department of Medical Illustrations, Makerere University Medical School, kindly prepared Fig. 1 to 3.

Supported in part by Contract Nos. PH-43-67-1343 and PH-43-67-47 from the National Cancer Institute, National Institutes of Health, United States Public Health Service, and the Research Grants Committee, Makerere University College, Kampala, Uganda.

### REFERENCES

Brown, R. S., Haynes, H. A., Foley, H. T., Godwin, H. A., Berard, C. W. and Carbone, P. P.—(1967) *Ann. intern. Med.*, **67**, 291.

Fahey, J. L. and McKelvey, E. M.—(1965) *J. Immun.*, **94**, 84.

Krant, M. J., Manskopf, G., Brandrup, C. S. and Madoff, M. A.—(1968) *Cancer, N.Y.*, **21**, 623.

Lamb, D., Pilney, F., Kelly, W. D. and Good, R. A.—(1962) *J. Immun.*, **89**, 555.

Landy, M. and Lamb, E.—(1953) *Proc. Soc. exp. Biol. Med.*, **82**, 593.

Lewis, M. G.—(1967) *Lancet*, ii, 921.

Lewis, M. G. and Kiryabwire, J. W. M.—(1968) *Cancer, N.Y.*, **21**, 876.

Smith, R. T.—(1968) *New Engl. J. Med.*, **23**, 1268.

Solowey, A. C. and Rapaport, F. T.—(1965) *Surgery Gynec. Obstet.*, **121**, 756.


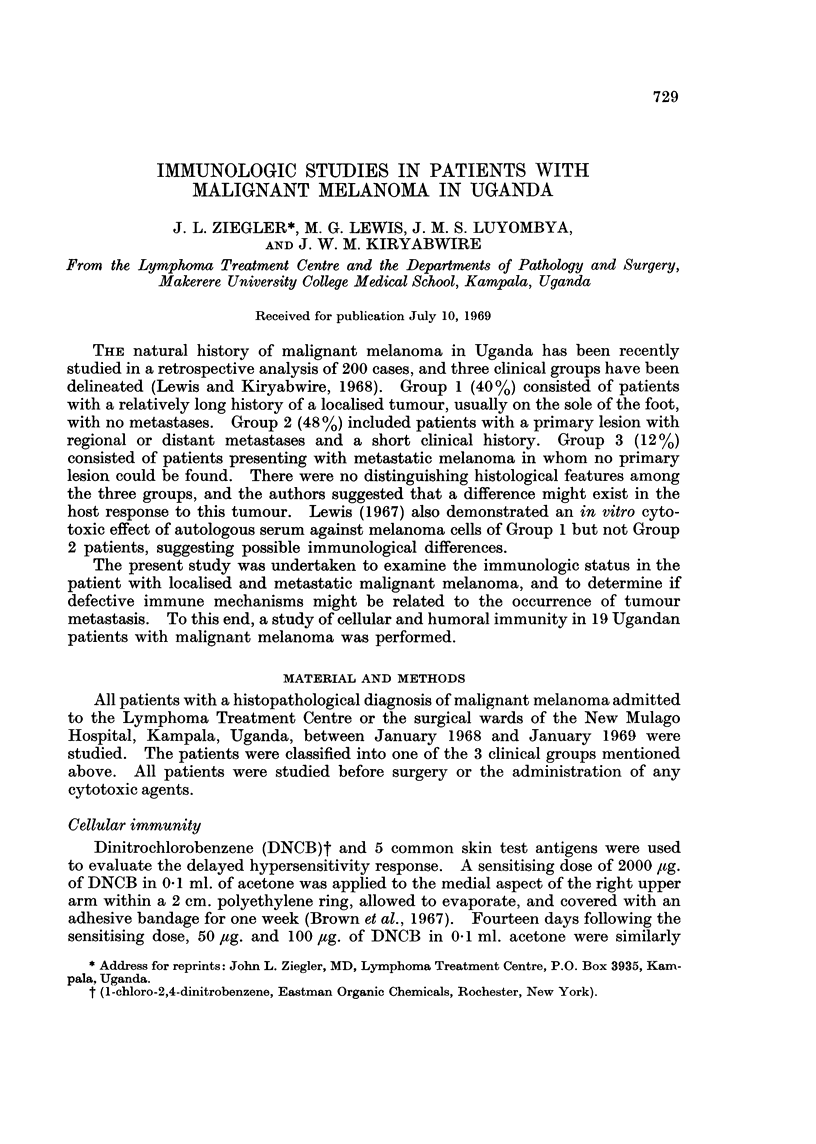

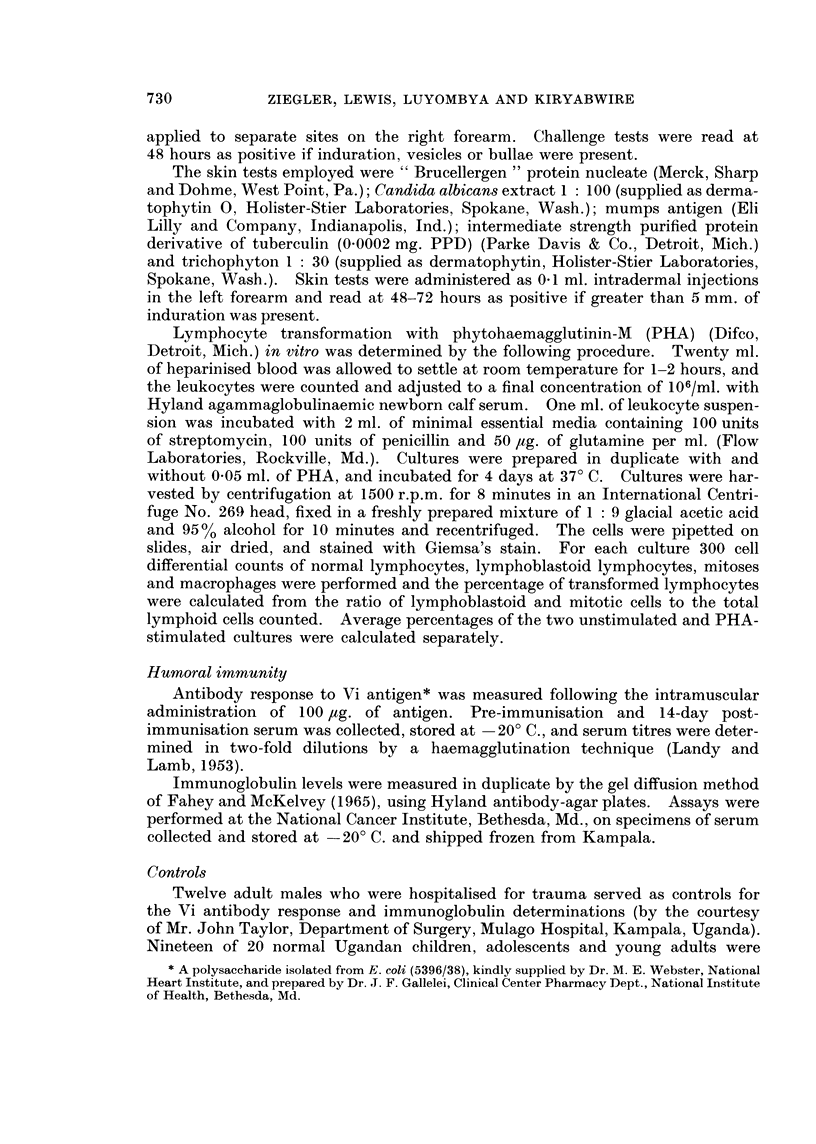

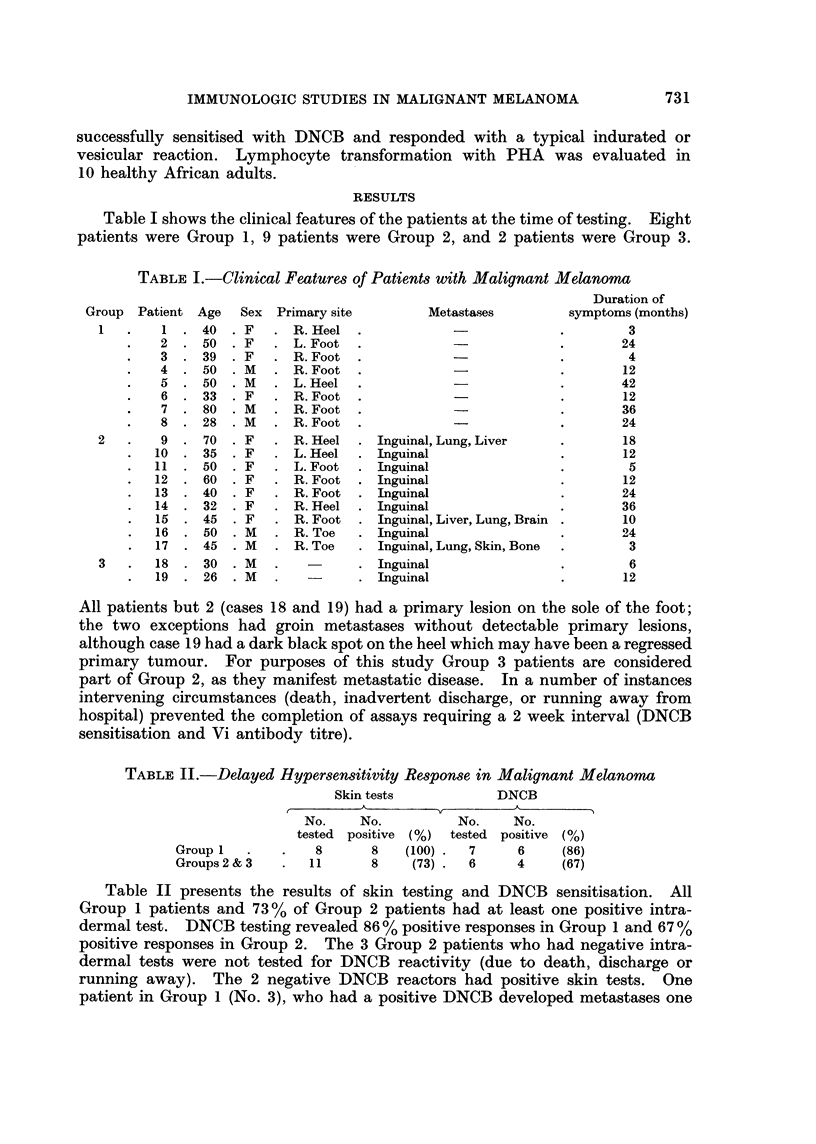

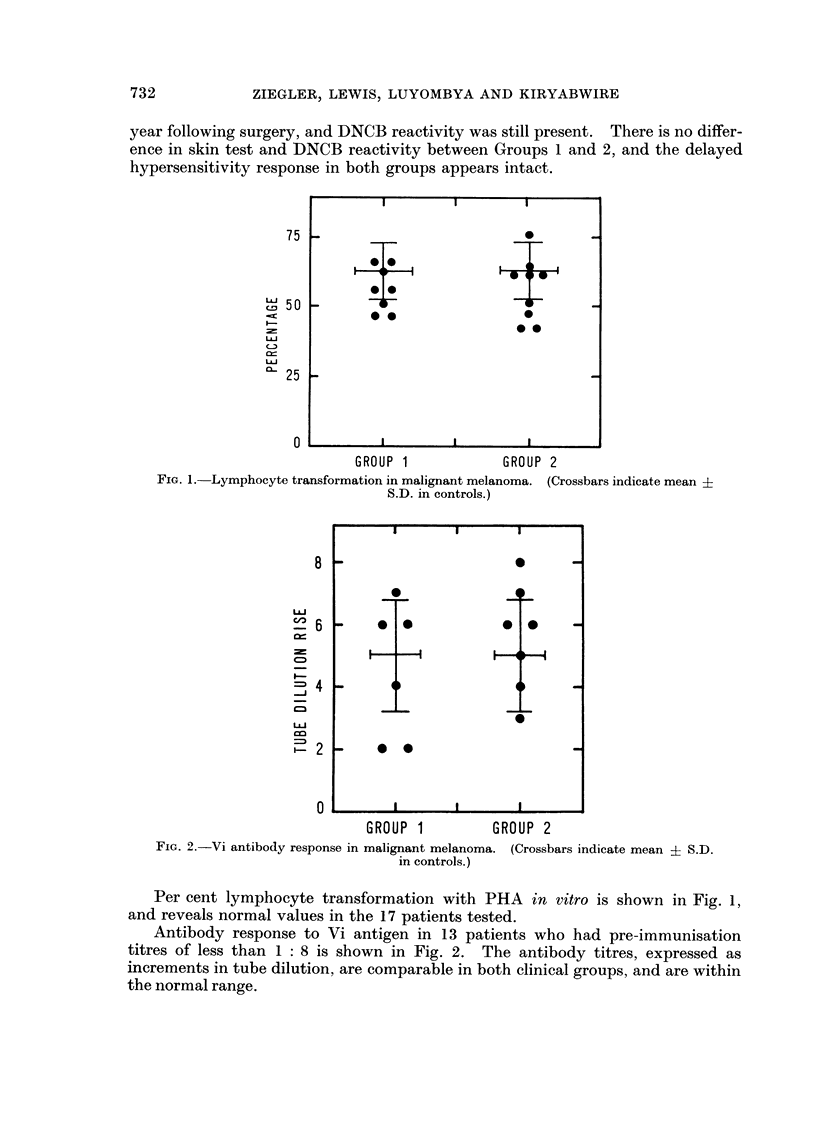

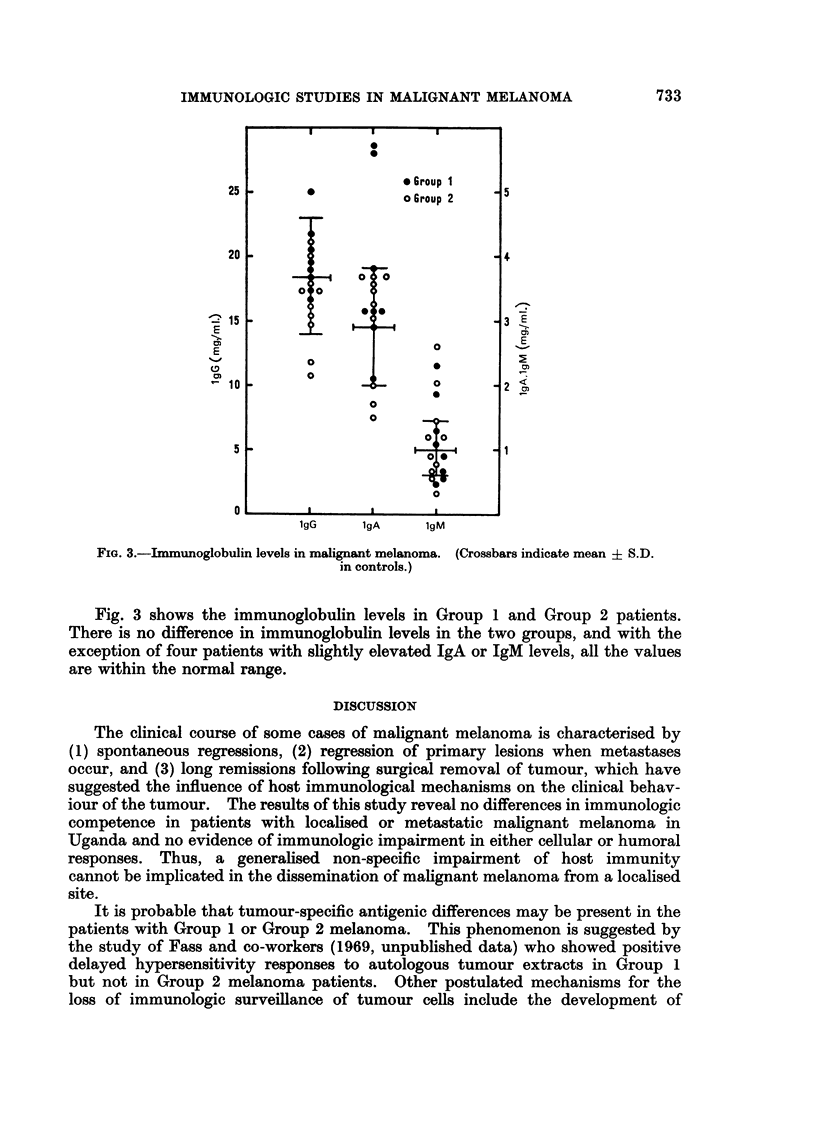

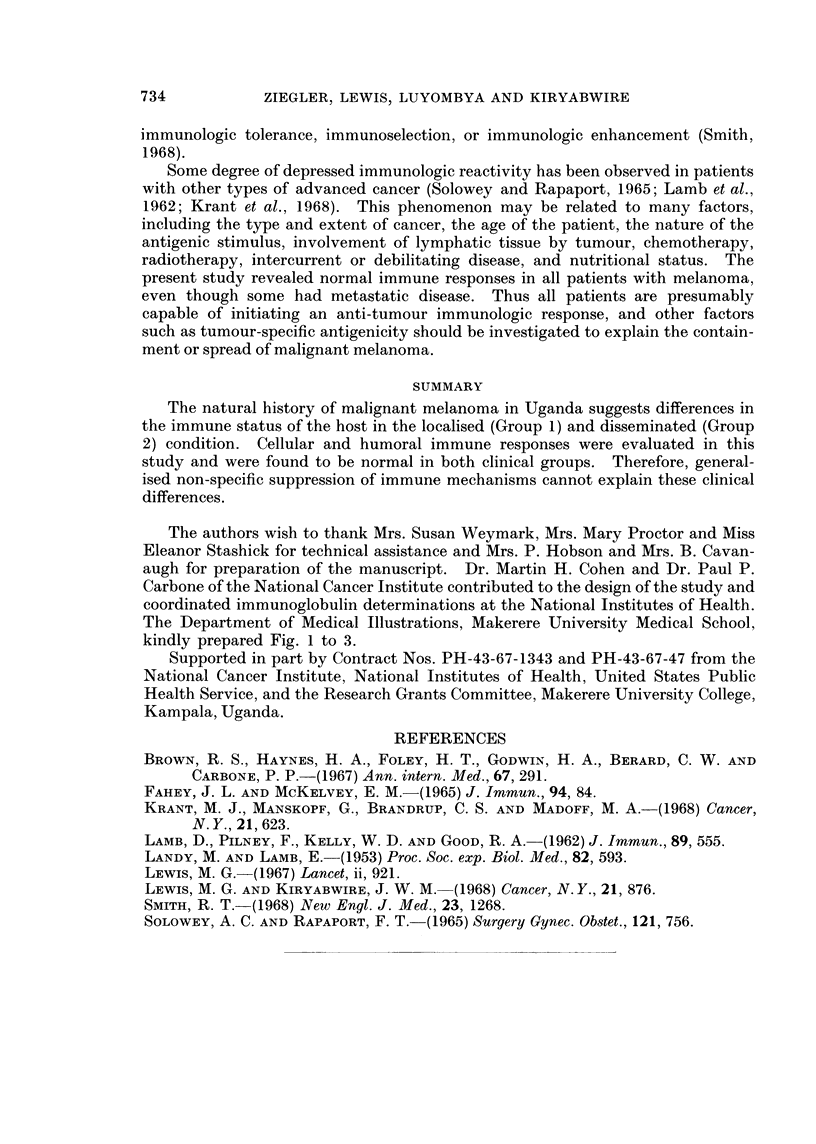


## References

[OCR_00421] Brown R. S., Haynes H. A., Foley H. T., Godwin H. A., Berard C. W., Carbone P. P. (1967). Hodgkin's disease. Immunologic, clinical, and histologic features of 50 untreated patients.. Ann Intern Med.

[OCR_00425] Krant M. J., Manskopf G., Brandrup C. S., Madoff M. A. (1968). Immunologic alterations in bronchogenic cancer. Sequential study.. Cancer.

[OCR_00429] LAMB D., PILNEY F., KELLY W. D., GOOD R. A. (1962). A comparative study of the incidence of anergy in patients with carcinoma, leukemia, hodgkin's disease and other lymphomas.. J Immunol.

[OCR_00430] LANDY M., LAMB E. (1953). Estimation of Vi antibody employing erythrocytes treated with purified Vi antigen.. Proc Soc Exp Biol Med.

[OCR_00433] Lewis M. G., Kiryabwire J. W. (1968). Aspects of behavior and natural history of malignant melanoma in Uganda.. Cancer.

[OCR_00436] Solowey A. C., Rapaport F. T. (1965). Immunologic responses in cancer patients.. Surg Gynecol Obstet.

